# Context-dependent activation of a social behavior brain network associates with learned vocal production

**DOI:** 10.21203/rs.3.rs-2587773/v1

**Published:** 2023-02-17

**Authors:** Katherine L. Anderson, Lionel Colón, Violet Doolittle, Raysa Rosario Martinez, Joseph Uraga, Osceola Whitney

**Affiliations:** The Graduate Center, CUNY; City University of New York, City College; City University of New York, City College; City University of New York, City College; City University of New York, City College; City University of New York, City College

**Keywords:** animal behavior, songbird, social behavior network, activity-dependent gene expression

## Abstract

In zebra finches, an avian brain network for vocal control undergoes context-dependent patterning of song-dependent activation. Previous studies in zebra finches also implicate the importance of dopaminergic input in producing context-appropriate singing behavior. In mice, it has been shown that oxytocinergic neurons originated in the paraventricular nucleus of the hypothalamus (PVN) synapse directly onto dopamine neurons in the ventral tegmental area (VTA), implicating the necessity of oxytocin signaling from the PVN for producing a context-appropriate song. Both avian and non-avian axonal tract-tracing studies indicate high levels of PVN innervation by the social behavior network. Here, we hypothesize that the motivation for PVN oxytocin neurons to trigger dopamine release originates in the social behavior network, a highly conserved and interconnected collection of six regions implicated in various social and homeostatic behaviors. We found that expression of the neuronal activity marker *EGR1* was not strongly correlated with song production in any of the regions of the social behavior network. However, when *EGR1*expression levels were normalized to the singing rate, we found significantly higher levels of expression in the social behavior network regions except the medial preoptic area during a social female-directed singing context compared to a non-social undirected singing context. Our results suggest neuronal activity within the male zebra finch social behavior network influences the synaptic release of oxytocin from PVN onto dopaminergic projection neurons in the VTA, which in turn signals to the vocal control network to allow for context-appropriate song production.

## Introduction

**Figure 1:**
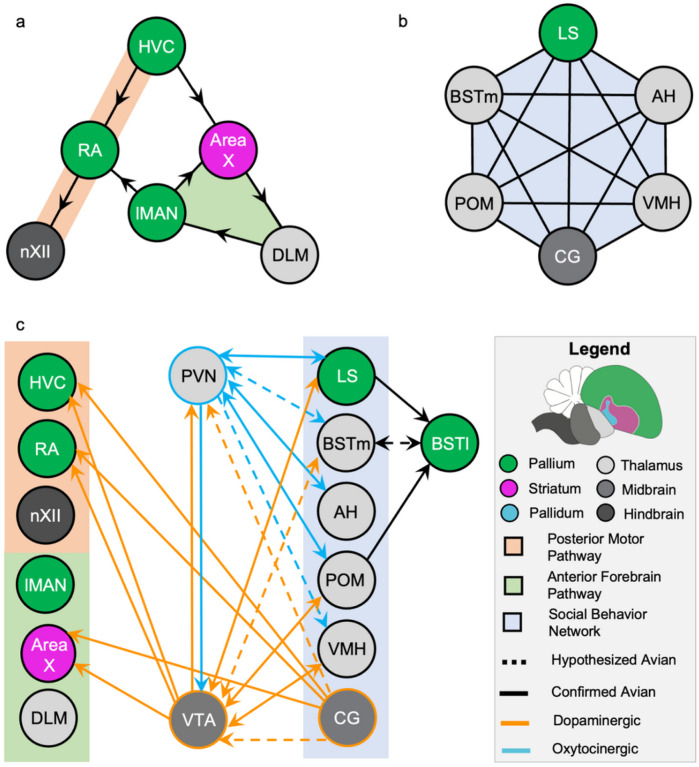
Axonal connections between avian neural networks for vocal learning and production and social behavior. The vocal control network (**a**) is comprised of two interconnected pathways: the posterior motor pathway (orange background; HVC, RA, and nXII) and the anterior forebrain pathway (green background; Area X, lMAN, and DLM). The social behavior network (**b**) is comprised of six interconnected regions (blue background; LS, BSTm, AH, POM, VMH, and CG). The brain area each region is located within is demonstrated by the color of the circle surrounding the region name, color key, and relative locations of each region are shown in the figure legend (green is pallium, pink is striatum, light gray is thalamus, medium gray is midbrain, and dark gray is hindbrain). In avian species, the social behavior network has direct and indirect connections to the vocal control network. These connections primarily work through the dopaminergic system (orange arrows), but the involvement of oxytocin is implicated as well (blue arrows). Solid lines represent the results of axonal tract-tracing studies performed in avian species, and dotted lines represent results from non-avian studies that the authors hypothesize are conserved in songbirds. Connections literature outlined in Table 1

Activation of a well-defined brain network in songbirds plans and initiates learned vocal behavior (Nottebohm et al. 1976; Long and Fee 2008). This network for learned vocal control in zebra finch songbirds (*Taeniopygia guttata*) comprises two neural circuits. A posterior circuit (HVC [proper name], robust nucleus of the arcopallium [RA], and tracheosyringeal subdivision of the hypoglossal nucleus [nXII]) projects to the vocal organ for song stereotypy while an anterior circuit (Area X [proper name], lateral magnocellular nucleus of the nidopallium [lMAN], and dorsolateral anterior thalamic nucleus [DLM]) allows for song plasticity and has a crucial role in vocal learning (Brainard and Doupe 2000; Kao et al. 2005). The posterior and the anterior neural circuits of this vocal control network are interconnected (Fig. 1a). Singing-dependent activation of this vocal control network can be assessed through the expression of activity-dependent genes, notably immediate early genes. (Jarvis and Nottebohm 1997; Jin and Clayton 1997; Kimpo and Doupe 1997; Jarvis et al. 1998, 2000; Whitney et al. 2014).

As adults, male zebra finches produce learned social female-directed songs to court potential mates, social juvenile-directed songs to tutor progeny, and social and non-social undirected song (Derégnaucourt et al. 2013; Chen et al. 2016). RA, Area X, and lMAN of the vocal control network show different activation patterns during social female-directed singing compared to non-social undirected singing (Jarvis et al. 1998). This social context-dependent difference in vocal control network activation is also observed through changes in vocal motor song output (Jarvis et al. 1998), as well as at the level of single neuron firing rates within the vocal control network (Kao et al. 2008). How external social contexts are conveyed to affect the activity of the vocal control network and subsequent singing behavior is not well understood, although current studies implicate dopaminergic influence (Sasaki et al. 2006; Kubikova et al. 2010; Ihle et al. 2015; Gadagkar et al. 2016).

Social context-dependent neural activation patterns of the vocal control network have been primarily attributed to dopaminergic inputs originating in the ventral tegmental area (VTA) (Heimovics and Riters 2005; Hara et al. 2007; Riters 2012). The VTA sends dopaminergic projections to Area X (Lewis et al. 1981; Reiner et al. 2004b; Castelino et al. 2007; Gale et al. 2008), HVC (Appeltants et al. 2000), and RA (Appeltants et al. 2002) in the vocal control network – of which Area X and RA show social-context-dependent cellular activation. The lack of context-dependent modulation of cellular activation in HVC, despite VTA-innervation, suggests a secondary method for conveying social stimuli presence. High levels of circulating oxytocin, a well-conserved nonapeptide that binds promiscuously to a family of G-protein-coupled receptors, correlate with increased social flocking behaviors in zebra finches (Goodson et al. 2009). Some suggest that oxytocin, primarily produced in the paraventricular nucleus of the hypothalamus (PVN), could serve as a neuromodulator of context-dependent behaviors.

Recent work in mammals has highlighted a mechanism for oxytocin-mediated social-context-dependent control of dopamine release from VTA. Oxytocin+ projection neurons, originating in the PVN, synapse directly and selectively onto dopaminergic neurons in the VTA (Hung et al. 2017; Xiao et al. 2017). VTA-projecting oxytocin neurons are more active following social encounters (Hung et al. 2017); subsequently, as oxytocin delivery to the VTA increases, so do the firing rates of dopamine neurons (Xiao et al. 2017). These dopaminergic neurons in the VTA that show oxytocin-mediated activity patterns express the oxytocin and vasotocin 1A receptors (Theofanopoulou et al. 2021). Both receptors are sensitive to oxytocin (Xiao et al. 2017). Altogether, these data suggest that during social encounters, oxytocinergic PVN to VTA projection neurons release oxytocin, which binds to either the oxytocin or vasotocin 1A receptors on dopaminergic VTA neurons, increasing neural firing rates in VTA. This cascade results in further signaling at the ultimate projection sites of these VTA dopamine neurons. Axonal tract-tracing studies performed in avian and mammalian species show that the PVN either uni- or bi-directionally connects with all regions of a brain network implicated in social behavior (Fig. 1c & Table 1). These data suggest that the strict control of synaptic oxytocin release may be dependent on signals originating in one or multiple regions of this social behavior network.

In vertebrates, many social behaviors, including those related to reproductive success, are regulated by the activity of six interconnected brain regions collectively referred to as the social behavior network (Newman 1999; Goodson 2005; O’Connell and Hofmann 2011; Kabelik et al. 2018; Eswine et al. 2019; Horton et al. 2020; Prior et al. 2021). This social behavior network is completely interconnected (Newman 1999). Defined regions of the avian social behavior network are in the cortex (lateral septum [LS]), the thalamus (medial bed nucleus of the stria terminalis [BSTm], medial preoptic area [POM], anterior hypothalamus [AH], and ventromedial hypothalamus [VMH]), and the midbrain (central gray [CG]) subregions of the brain (Fig. 1b). The involvement of the social behavior network in species-Specific adaptive, mainly courtship, behaviors seem to be conserved across vertebrates. Specific analyses of this network have been performed in rodents (Wood and Newman 1995; Newman 1999), amphibians (Laberge et al. 2008), reptiles (Sakata et al. 2000; Sakata and Crews 2004; Kabelik et al. 2018), fish (Goodson and Bass 2002), and birds (Edwards et al. 2020). Additionally, the chemo-architecture of the social behavior network shows conservation across vertebrate species (Goodson et al. 2004; Kingsbury et al. 2011). Regions of the social behavior network also include continuous subdivisions of the extended amygdala (Johnston 1923a; Olmos and Ingram 1972). The mammalian extended amygdala (lateral bed nucleus of the stria terminalis [BSTl], BSTm, & medial amygdala) is implicated in managing emotional responses to external stimuli. (Johnston 1923a; Olmos and Ingram 1972). Strong connections to the medial amygdala may prime the social behavior network to deliver the emotional relevance of external stimuli to other networks within the brain in a context-dependent manner. The potential integration of the social behavior network to other defined neural systems is not well established (Kelly 2022). Nonetheless, the involvement of the social behavior network in motivating the context-appropriate performance of learned behaviors is highly plausible.

We hypothesize that the social behavior network may send context-Specific information to PVN, facilitating oxytocin-dependent activation of dopaminergic neurons within VTA, which in turn enables context-appropriate song production and neural activation in the vocal control network of adult male zebra finches. In the present study, we test this hypothesis by assessing whether activation patterns of the social behavior network are different during the performance of a learned behavior in non-social and social contexts. Using brain tissue from adult male zebra finches performing either non-social undirected songs or social female-directed songs, we analyzed the transcription of activity marker *EGR1* using fluorescence *in situ* hybridization. We found upregulation of *EGR1* mRNA in the AH, BSTm, CG, LS, and VMH of the social behavior network, the BSTl, and the PVN during social singing contexts compared to non-social singing contexts. These data provide correlational evidence that the social behavior network does play a role in the differential production of female-directed versus undirected songs in zebra finches.

## Methods

### Animal husbandry

All experimental animals and techniques were approved by the City College of New York (CCNY) Institutional Animal Care and Use Committee and met state and federal standards for animal care and welfare in accordance with the guidelines of the National Institute of Health. Zebra finches were bred and housed at the CCNY Marshak vivarium on a 14:10 hour light: dark cycle with *ad libitum* access to seed, water, and cuttlebone. Birds were also supplementally fed egg and millet twice a week. The sex of the birds as juveniles was determined using standard PCR (Soderstrom et al. 2007) and confirmed visually in adults using plumage. Behavioral experiments with adults (> 120 days old) were performed at the CCNY Center for Discovery and Innovation, where animals were similarly housed in sound-attenuating chambers. All experimental birds were acclimated to sound-attenuating chambers for a minimum of two days before behavioral tests were conducted.

### Behavioral conditions and analysis; tissue collection and preparation

All behavioral observations occurred in a sound-attenuating chamber. Adult males were observed either in the acoustic and visual presence of an adult female (n = 5) or in isolation (n = 10). All animals were maintained on a long photoperiod that simulates a single season year-round. Zebra finches are opportunistic breeders, not seasonal breeders. Therefore seasonal hormone fluctuations were not considered to impact individual singing rates and were not measured. After 45 minutes of observation, beginning either at the start of the light cycle (9:30 AM) or the onset of singing behavior, animals were sacrificed via rapid decapitation. All animals were captured between 10:15 AM and 10:45 AM. Brains were quickly excised from the skulls, flash-frozen in cryoprotective molds, and stored at −80° C until future use. Fresh-frozen tissue was coronally sectioned at 8 μm using a cryostat (CM1950, Leica Biosystems, Deer Park, IL, USA) and thaw-mounted onto charged glass slides in series. Tissue slides were kept at −80° C for storage until future use. All song bouts produced during the observation window were recorded using Sound Analysis Pro 2011 (Tchernichovski et al. 2000). After sacrifice, the total seconds of song produced by each animal was manually calculated using Avisoft Bioacoustics Sound Analysis and Synthesis Software (Glienicke/Nordbahn, Germany). To determine the singing rate based on the number of song bouts produced, the total number of song bouts was divided by the time in minutes between the first bout of the observation period and the time of sacrifice. In addition, the individual singing rate for each animal was analyzed as seconds of singing per minute of observation by dividing the total seconds of singing by the time in minutes between the first bout of the observation period and the time of sacrifice. To avoid dividing by zero when normalizing expression rated in non-singing animals, all singing rates were increased by 1.

### Molecular cloning

Our isolated *EGR1* sequence from zebra finch brain tissue was comparable to NCBI transcript accession number NM_001080957.1. To clone *EGR1*, total RNA was isolated from the whole brains of adult male zebra finches using a tissue homogenizer and the Promega SV Total RNA Isolation System (Z3100, Promega, Madison, WI, USA). To synthesize cDNA, whole brain total RNA was used with oligo dT_20_ primers from the Invitrogen SuperScript IV First-Strand Synthesis System (Thermo Fisher Scientific, Waltham, MA, USA). *EGR1* transcripts were isolated from cDNA via PCR using zebra finch-Specific EGR1 forward and reverse primers (respectively, 5’-TGCAGATCTCCGACCCCTTTG and 5’-GGATCAGCAGATCTCAATTGTCC). An amplified transcript of the expected size was gel-extracted and ligated into a pGEM-T Easy Vector (A137A, Promega, Madison, WI, USA) at a 3:1 insert: vector molar ratio. The *EGR1*-containing plasmid vector was amplified further after transfection into JM109 competent *E*. *coli* cells (L2005, Promega, Madison, WI, USA). Plasmids containing EGR1 were purified from bacterial cultures using the ZymoPURE Plasmid Miniprep kit (D4210, Zymo Research, Irvine, CA, USA) and stored at −20° C until further use. The plasmids were Sanger sequenced in both forward and reverse directions to confirm the identity of the transcript.

### mRNA antisense probe transcription

All antisense mRNA probes were generated in a two-hour transcription reaction at 37°C. Template DNA for this transcription reaction was made through a polymerase chain reaction using M13 forward and reverse primers and plasmids. Transcription reaction components were as follows: 1000 ng template DNA, 1 μL of RNase inhibitor (03335399001, Millipore Sigma-Aldrich, Burlington, MA, USA), 2 μL of T7 RNA polymerase (10881767001, Millipore Sigma-Aldrich, Burlington, MA, USA), 2 μL of 10X FITC-dUTP labeling nucleotides (11685619910, Millipore Sigma-Aldrich, Burlington, MA, USA), and 2 μL of 10X transcription buffer (11465384001, Millipore Sigma-Aldrich, Burlington, MA, USA). The reaction volume was adjusted to 20 μL using molecular biology-grade water. To halt the transcription reaction, 2 μL of DNase I (101228–386, VWR, Radnor, PA, USA) was added. To precipitate the RNA, 100 μL of TE buffer, 12 μL of 3M sodium acetate pH 5.2 (10128–584, VWR, Radnor, PA, USA), 400 μL of 100% ethanol, and 1 μL of linear polyacrylamide (AAJ67830-XF, VWR, Radnor, PA, USA)) were added to the reaction tube before incubating at −80° C for 45 minutes. RNA was pelleted by centrifuging at 4° C at 13.3 RPM for ten minutes. The supernatant was removed, and the pellet was washed using 70% ethanol. RNA pellet was resuspended in 10 μL of deionized water and 90 μL of deionized formamide. To prevent freeze-thawing, 5 μL aliquots were stored at −80° C until use.

### In situ hybridization

Tissue slides were fixed in 4% paraformaldehyde in 1X PBS for five minutes. The tissue slides were acetylated using acetic anhydride in TEOA for ten minutes, dried gradually using a graded series of ethanol washes (70%, 95%, 100%), and then left to dry at room temperature for one hour. Tissue slides were incubated overnight with *EGR1* dUTP-FITC-labeled antisense mRNA probes at a 1:100 dilution in a hybridization buffer containing 300 mM NaCl, 20 mM Tris-HCl, 5 mM EDTA, 10 mM PBS, 10% dextran sulfate, 500 ug/mL tRNA, 200 μg/mL herring sperm DNA, and deionized formamide. The mRNA probes were allowed to hybridize overnight in a 64° C mineral oil bath to ensure even temperature distribution. Oil was removed from slides using chloroform washes after hybridization. Non-Specific hybridization was removed using 5X and 0.2X saline sodium citrate buffer washes at 64° C. Endogenous peroxidases were quenched using 3% hydrogen peroxide in 1X PBS prior to the addition of anti-fluorescein-POD at 1:1000 (11426346910, Millipore Sigma-Aldrich, Burlington, MA, USA) in a high-tris TBS and sheep serum blocking buffer. FITC was amplified using FITC-TSA at 1:100 (SAT701001EA, Akoya Biosciences, Marlborough, MA, USA). Then Alexa Fluor 488-anti-FITC at 1:500 (200-542-07, Jackson ImmunoResearch, West Grove, PA, USA), delivered overnight in Roche blocking buffer (11093274910, Millipore Sigma-Aldrich, Burlington, MA, USA). Slides were coverslipped out of deionized water using VectaShield with DAPI (H-1500, Vector Labs, Newark, CA, USA).

### Microscopy and image analysis

Images of resulting fluorescence in areas of interest were captured at 40X magnification in 488 nm (green) and 405 nm (DAPI) channels using an Olympus BX53 microscope and an Olympus DP74 camera (Shinjuku-ku, Tokyo, Japan). Fields of interest in each image were located based on established brain region delineations (Reiner et al. 2004b, a; Nixdorf-Bergweiler and Bischof 2007; Jarvis et al. 2013) in combination with tyrosine hydroxylase immunofluorescence images of the social behavior network in zebra finches (Goodson 2005). The FIJI ImageJ2 cell counter analysis plugin for macOS software was used to perform image quantification (Rueden et al. 2017). To determine the percentage of EGR1 + cells per field of interest, the number of EGR1 + cells was divided by the number of DAPI + cells in the same field. Each field of interest was captured once per individual. No hemiparetic differences in *EGR1* expression were detected visually for any region, so the data from each hemisphere were aggregated. Cell counts were independently replicated four times for each region of interest, and the percentage of EGR1 + cells was averaged together for each bird.

### Data analysis and visualization

A two-way Analysis of Variance (ANOVA) test was run to determine significant sources of variation between a brain region and social context. In addition, a Šidák’s multiple comparisons test was used to determine significant differences in cellular activation within each region of interest when compared to the same region in different experimental contexts. P-values less than 0.05 were considered significant. Data and graphs were analyzed and generated using GraphPad Prism version 9.3.0 (345) for Mac OS X (GraphPad Software, San Diego, California, USA).

## Results

### Social behavior network cellular activation correlates with singing rate

Zebra finches were housed either in proximity to a female con Specific as a social context or alone as a non-social context. To quantify the singing amount of zebra finches in each social context, the seconds of singing and number of bouts produced during the observation period were recorded and compiled manually. Other than being exposed to a particular social context, none of the birds were provoked to sing or remain silent during the behavioral observation period. Lower singing rates were recorded from males singing in proximity to females compared to males that chose to sing in isolation. Seconds singing ranged from 0–43.73 seconds in social males and from 0–375.48 seconds in isolated males. Social males in this study produced up to 21 bouts, while males who chose to sing in isolation produced up to 183 bouts. Bout length averages across all individuals were between 1.12–4.76 seconds.

**Figure 2:**
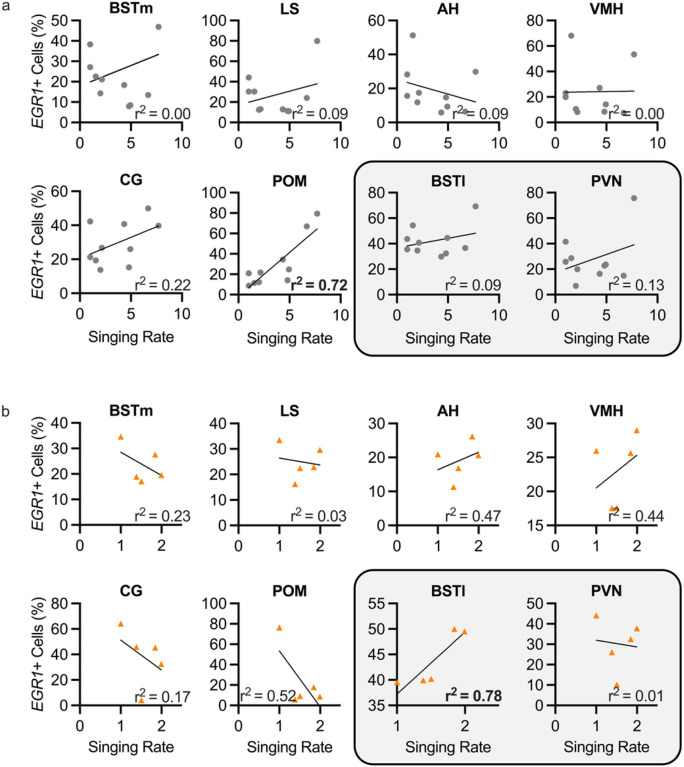
Correlations between singing rate and percentage of cellular activation in all regions of the social behavior network (including the BSTl) and the PVN in non-social and social conditions. Percentage of cells expressing EGR1 is plotted against the number of song bouts produced for individual animals in a non-social undirected singing condition (**a**, gray circles) and a social female-directed singing condition (**b**, orange triangles) for all regions of the social behavior network (including the BSTl) and the PVN. Low correlations (r^2^ < 0.52) were seen for all regions in non-social and social contexts except for the POM in non-social singing contexts and the BSTl in social singing contexts, which had r^2^ values of 0.7221 and 0.7794, respectively. Significant r^2^ values are bolded

To determine if the cellular expression of *EGR1* mRNA in regions of the social behavior network, the BSTl, and the PVN could be related to singing amount, we plotted the correlation between the percentage of *EGR1* expression in those regions and the singing rate as either seconds spent singing per minute of observation or the number of song bouts produced per minute of observation. We found the strongest correlations between cellular *EGR1* expression and the seconds-based singing rate (Fig. 2) compared to correlations between *EGR1* expression and the bouts-based measure of singing rate (Supplemental Fig. 1). For all eight regions examined, correlations between cellular *EGR1* expression and the number of bouts singing rate produced showed no significant r^2^ values in non-social and social contexts (range r^2^ = 0.00–0.70). However, four regions of the social behavior network showed a moderately high correlation between *EGR1* cellular activation and the seconds of singing rate in social contexts (range r^2^ = 0.23–0.52). In addition, two regions of the highly interconnected social behavior network showed a significant correlation between *EGR1* cellular activation and the seconds of singing rate. The POM in non-social singing contexts and the BSTl in social singing contexts had r^2^ values of 0.7221 and 0.7794, respectively (Fig. 2). These data suggest a role for the social behavior network, and particularly the POM and the BSTl, in mechanisms for motivating the production of undirected and female-directed song, respectively.

### Social-context-dependent activation of the social behavior network during performance of learned vocalizations

**Figure 3:**
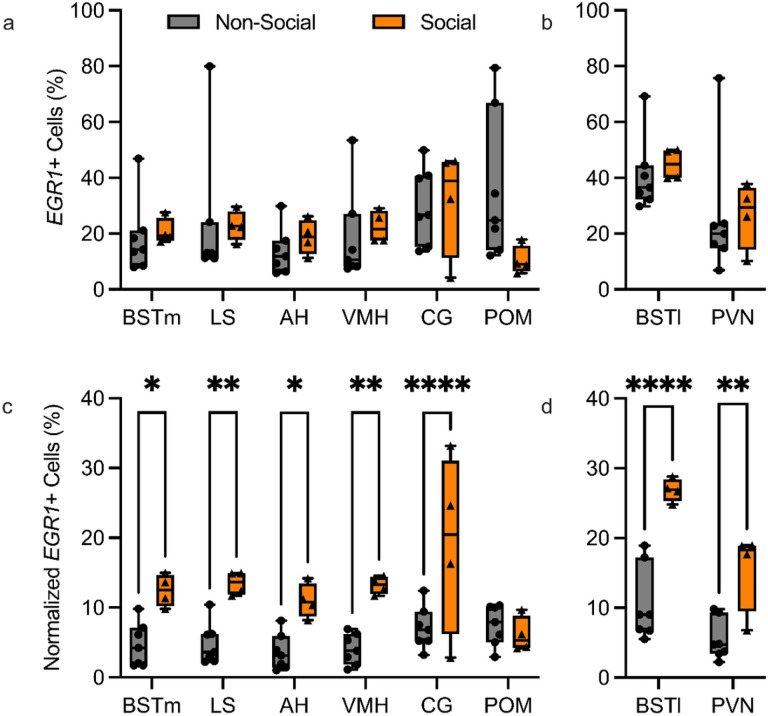
Cellular activation within each region of the social behavior network (including the BSTl) and the PVN in non-social and social conditions. Cellular activation is plotted both unnormalized (**a** & **b**) and normalized to singing rate (**c** & **d**). No significant differences were found for unnormalized cellular activation in non-social (gray bars, circle icons, n=10) versus social (orange bars, triangle icons, n=4) singing conditions. After normalization to the singing rate, all regions except the POM showed higher levels of cellular activation in social singing conditions compared to non-social singing conditions. Each icon represents an individual animal, and each region was analyzed in every animal included within a single context. *= p-value < 0.05, ** = p-value < 0.01, *** = p-value < 0.001, **** = p-value < 0.0001

**Figure 4:**
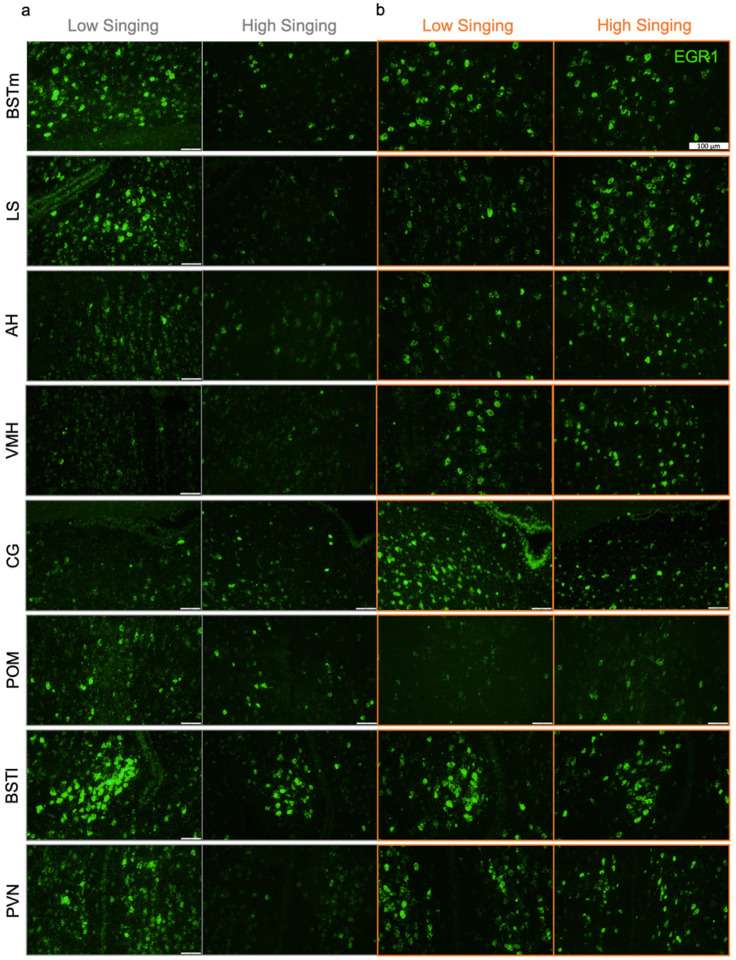
Representative fluorescent *in situ* hybridization images for all regions of the social behavior network (including the BSTl) and the PVN in non-social and social conditions. 40X fluorescent images are presented for adult male zebra finches performing low (under one bout per minute) and high (over one bout per minute) singing amounts in non-social undirected (**a**) and social female-directed (**b**) contexts. Counts were performed only in regions of interest and did not include cells from surrounding brain areas. Region boundaries were hand drawn based on borders shown in previous literature. Scale bar represents 100 μm

To assess the singing-independent differences in *EGR1* mRNA expression within all regions of interest in non-social undirected and social female-directed singing conditions, we plotted the percentage of cells expressing *EGR1* for each region of interest in each social context. High levels of variation were observed in the percentage of cells expressing *EGR1* for each region of the social behavior network across individuals within both non-social and social singing conditions (Fig. 3a). The same trend was also observed for the BSTl and PVN (Fig. 3b). No significant singing-independent social-context-dependent difference in *EGR1* mRNA expression was found for any of these regions. Representative cellular labeling for *EGR1* in each region in non-social (Fig. 4a) and social (Fig. 4b) adult male birds are provided. These results suggest that social context alone does not explain the differences in the induction of cellular *EGR1* expression in the social behavior network (including the BSTl) and the PVN.

Finally, to assess song-dependent differences for each social context, we evaluated the normalized *EGR1* expression in each region of interest based on the singing rate as time (in seconds) spent singing. All regions of the social behavior network except for the POM showed upregulated *EGR1* expression in social female-directed singing adult males compared to non-social undirected singing males (Fig. 3c). BSTl and PVN also showed similarly increased cellular EGR1 mRNA expression levels when normalized to singing rate (Fig. 3d). Additionally, variation in *EGR1* cellular expression across individuals within a single experimental category was strikingly reduced in the singing rate normalized data compared to non-normalized values in nearly all regions and contexts. These data implicate the involvement of the social behavior network (including the BSTl) and the PVN in the neural mechanisms underlying the motivation to sing. In female-directed singing male CG, greater variation in *EGR1* expression across individuals, even after normalization to singing, suggests that, in social contexts, the CG receives or sends signals more dependent on individual experiences.

## Discussion

Healthy social interactions require some form of vocal behavior for many species. In frogs, the BST appears crucial for evaluating social cues and initiating vocalizations (Hall et al. 2013; Kwong-Brown et al. 2019). Tokay geckos, in the presence of broadcast noise compared to quiet conditions, increase their brief call note duration and produce more high-amplitude syllable types, which facilitates signal detection by conSpecific receivers (Brumm and Zollinger 2017). Multiple bird species are inherently social (Csillag et al. 2022), and songbirds, such as the zebra nch, can identify conSpecific individuals based on unique vocal signatures (Elie and Theunissen 2018). Bottlenose dolphins use their learned whistles in matching interactions to address each other for affiliative purposes (Janik 2000; Chereskin et al. 2022). In other species, such as seals, where rapid mother-young social recognition is crucial for offspring survival, postnatal individual vocal recognition can occur within hours (Martin et al. 2022). The highly organized social structure of the naked mole rat is maintained using the social transmission of vocal dialects (Barker et al. 2021). In the primate brain, neural representations of social vocal signals are highly flexible and appear to reflect the nuances of dynamic behavioral contexts (Jovanovic et al. 2022). These investigations, as well as many others, including in mice (Arriaga and Jarvis 2013), indicate that social vocal behavior is a fundamental part of social behavior across vertebrate amphibians, reptiles, birds, and mammals (Chen and Wiens 2020). Moreover, research on social vocal behavior has revealed both the convergence and divergence of brain circuits for vocal production (Jarvis 2019; Kelley 2022). One clear outcome of this work is that the brain architectures for non-learned and learned vocal behavior are fundamentally different. In addition, these studies also indicate that social vocal behavior requires coordination between brain regions integrating internal and external information and brain regions for vocal behavior action. Nevertheless, the brain circuits that integrate such information to motivate an appropriate learned behavioral action are not well established.

Vocal control regions in zebra finches show patterns of differential cellular activation during social versus non-social song production. This previous knowledge, along with other experimental investigations of the involvement of the social behavior network in vertebrate adaptive behaviors, led us to inquire whether the social behavior network may influence the social-context-dependent song production in adult male zebra finches. Here we investigate in adult male zebra finches whether neural activity in the social behavior network is related to the production of social-context-appropriate singing behavior. Using brain tissue captured immediately after a 45-minute observation, we assessed neuronal activation within the social behavior network in either non-social (alone) or social (paired with a female) contexts. We examined the expression of activity marker *EGR1* in several regions of the social behavior network, which included the AH, BSTl, BSTm, CG, POM, and VMH, and in an oxytocin production region, the PVN. The PVN is bidirectionally connected to the VTA, a major site for dopamine production. The expression of *EGR1* cellular activity was normalized to the song production rate as time spent singing. Across all regions except for the POM, we found increased levels of song-normalized cellular activity from singing males in a social context compared to males who sang in a non-social context.

We found stronger correlations between cellular activation in the social behavior network and a singing time-based measure of song production rather than a song bout-based measure. Previous literature found that the number of bouts produced had a stronger relationship with *EGR1* expression in the vocal control network compared to seconds spent singing (Jarvis et al. 2000). These data may suggest that cellular activation of the social behavior network, including the BSTl and the PVN, in relation to song production is under different temporal dynamics than that of the vocal control network with regard to singing behavior. Furthermore, these results suggest that while activation of the social behavior network seems to be correlated with song production, this activation is not singing-dependent but motivates social context-dependent singing behavior.

The strength of the correlations between cellular activation and the singing rates for each individual differentially implicates regions of the social behavior network for motivating the production of a social-context-appropriate learned behavior. In social conditions, a strong positive correlation was found between singing rate and cellular activation of the BSTl. This finding suggests that particularly salient social stimuli transmitted by the BSTl to the rest of the social behavior network could substantially drive a subsequent increase in song production. In non-social contexts, POM showed a strong positive relationship between singing rate and cellular activation. Previous investigations in a seasonal breeding songbird report an increase in non-female-directed singing following POM inactivation (Alger and Riters 2006). These findings and the results of our present investigation suggest that inactivating any single region of the social behavior network could lead to compensatory changes due to the highly interconnected nature of the network. Additional studies involving the manipulation of any one region of the social behavior network should consider analyzing subsequent changes in all the connected regions.

Overall, our results support a hypothesis that social context modulates the activity of an interconnected social behavior network of forebrain regions within the hypothalamus that promotes adaptive behavioral responses. Furthermore, our experiments in zebra finches establish a functional connection between these hypothalamic regions of the social behavior network, an oxytocin-releasing brain region, and the vocal control network. Differential activation of these hypothalamic regions could act as a decision-making network that modulates oxytocinergic input to the mesostriatal dopamine system to motivate context-appropriate learned vocal production.

## Figures and Tables

**Table 1.** Afferent and efferent projections between BSTl, PVN, and VTA and the social behavior network.

Nuclei	Afferent Projections			Efferent Projections		
		Aves	Non-Aves		Aves	Non-Aves
**BSTI**	**BSTm**		1,2	**BSTm**		1,2
	**LS**	3		**PVN**		4–6
	**POM**	7				
	**PVN**		8			
**PVN**	**AH**		4, 8, 9	**AH**	10	8, 11
	**BSTm**		6	**BST**		8
	**CG**		12	**LS**		8
	**LS**	3, 13		**POM**	14	8, 15
	**POM**	7, 10	4, 7, 9, 10, 15	**VMH**		8, 16, 17
	**VTA**	13		**VTA**	13	27–29
**VTA**	**BST**		29	**BSTm**		20
	**CG**		18, 19	**LS**	13	22, 30
	**LS**		21, 29	**POM**	7, 10	11, 24–26
	**POM**	7, 10, 23	15	**PVN**	13	
	**PVN**	13	29	**VMH**	10	
	**VMH**	10				

In addition to intra-network axonal projections, regions of the social behavior network receive and make afferent and efferent axonal projections from and to regions outside this network. References to previous tract-tracing experiments are shown for avian and non-avian species. 1 = (Johnston 1923b); 2 = (Olmos and Ingram 1972); 3 = (Montagnese et al. 2004); 4 = (Sawchenko and Swanson 1983); 5 = (Otake and Nakamura 1995); 6 = (Dong et al. 2001); 7 = (Riters and Alger 2004); 8 = (Pfaff and Conrad 1978); 9 = (Prewitt and Herman 1998); 10 = (Balthazart et al. 1994); 11 = (Delville et al. 2000); 12 = (Li et al. 2014); 13 = (Korf 1984); 14 = (Absil et al. 2002); 15 = (Simerly and Swanson 1988); 16 = (Fahrbach et al. 1989); 17 = (Shimogawa et al. 2015); 18 = (Omelchenko and Sesack 2010); 19 = (Ntamati et al. 2018); 20 = (Veening et al. 1982); 21 = (Meibach and Siegel 1977); 22 = (Swanson and Cowan 1979); 23 = (Iyilikci et al. 2017); 24 = (Haglund et al. 1984); 25 = (Risold and Swanson 1997); 26 = (Cádiz-Moretti et al. 2016); 27 = (Hung et al. 2017); 28 = (Xiao et al. 2017); 29 = (Beier et al. 2015); 30 = (Aransay et al. 2015)

## Data Availability

The datasets generated and analyzed during the current study are available from the corresponding author upon reasonable request.
